# Free-Standing Complementary Asymmetric Metasurface for Terahertz Sensing Applications †

**DOI:** 10.3390/s20082265

**Published:** 2020-04-16

**Authors:** Fatima Taleb, Ibraheem Al-Naib, Martin Koch

**Affiliations:** 1 Faculty of Physics, Philipps-Universität Marburg, Renthof 5, 35032 Marburg, Germany; martin.koch@physik.uni-marburg.de; 2 Biomedical Engineering Department, College of Engineering, Imam Abdulrahman Bin Faisal University, Dammam 31441, Saudi Arabia; iaalnaib@iau.edu.sa

**Keywords:** free-standing, asymmetric split-ring resonators, sensing, terahertz

## Abstract

We designed and tested a highly sensitive metasurface device based on free-standing complementary asymmetric split-ring resonators at terahertz frequencies. It is utilized for sensing a galactose film. We characterized the device using the induced red shift of a Fano resonance observed in the THz transmission. The sensor has a high sensitivity of 91.7 GHz/RIU due to a significant interaction between the galactose overlayer and the metasurface.

## 1. Introduction

The interaction of electromagnetic waves (EM) with metasurfaces has attracted tremendous attention [1,2], as it can be utilized for different applications such as the sensing of unidentified analytes [3,4,5]. Any modification in the permittivity and/or permeability of the sensor environment leads to a clear signature in the EM response. Many reported metasurfaces are promising for the design of devices with exotic electromagnetic properties [6,7,8,9,10]. Periodic resonant structures, with subwavelength dimensions, are the key in the design of metasurfaces. The optical properties of metasurfaces are tailored to represent the shape and the size of a resonant structure within the unit cell. Furthermore, a coupling between different unit cells exists. This allows for custom-made dielectric and/or magnetic properties within a given frequency range [11,12,13]. The salient applications of metasurfaces are negative refractive index, cloaking, super lenses, filtering and sensing [14,15,16,17,18,19,20,21].

Terahertz molecular sensors have gained great attention due to their distinctive characteristics and molecular signature in the terahertz frequency range [22,23,24]. For example, a metasurface with a double split-ring resonator (DSRR) as a unit cell has been used for detecting two monosaccharide molecules: glucose and galactose [25]. Besides, a nano-slot antenna array based sensing chip has been used for identifying different types of carbohydrate molecules and biomaterials such as viruses over a wide range of concentrations in the terahertz frequency range [26,27]. Moreover, many configurations have been recently proposed for terahertz thin-film sensing applications [28,29,30,31]. Most of those metasurface sensors are fabricated using photolithography, which is expensive and cumbersome. The metallic resonators are then positioned on a dielectric substrate [32]. However, this kind of configuration leads to a reduction in the sensitivity, as the field confinement takes place inside the dielectric substrates with a refractive index greater than one. This reduces the interaction between the deposited overlayer and the terahertz waves. Additionally, substrates with residual absorption can lead to losses. Hence, extremely thin substrates or membranes are used to enhance the sensitivity of the SRR sensors and to increase the frequency shift [33,34]. Such thin substrates clearly cause great fabrication and stability obstacles [35,36,37]. Furthermore, it was found that dielectric substrates cause a saturation in the frequency shift when the analyte reaches a thickness of a few micrometers. This reduces the effective volume of the probed analyte [38].

Metasurfaces can suffer from radiative losses. This can be mitigated by designing the metallic structure of metasurfaces such that it exhibits a sharp asymmetric resonance, which is known as Fano resonance [39,40,41]. Split-ring resonators show Fano resonances if their structural symmetry is broken [42,43,44]. This resonance can be spectrally sharp and can reach high quality factors. It is highly sensitive to minor changes in the surrounding environment. The radiative loss is consequently alleviated as a result of weak coupling of the radiation to free space [45,46].

In this work, we fabricate planar free-standing complementary asymmetric split-ring resonator (FCA-SRR) metasurface structures based on Babinet’s principle. This structure is realized by laser beam machining [47]. Hence, there is no need for a supportive dielectric substrate. Consequently, there is no field inside the substrate, and terahertz waves interact efficiently with the analyte. The absence of the substrate ensures that there is no dispersion or losses. This offers the possibility to construct a high-performance sensor. This device is then used for the recognition of two types of sugar molecules. The first one is sucrose (molecular formula C_12_H_22_O_11_), a disaccharide molecule that is a combination of two monosaccharides: glucose and fructose. The second one is galactose (C_6_H_12_O_6_), which is a monosaccharide molecule and a component of blood cell antigens. The importance of galactose arises from its impact on metabolism, senescence (aging) and the treatment of kidney disease [48]. Furthermore, our sensor allows for the detection of galactose over a wide range of concentrations. This manuscript is an extension version of the conference paper [49].

## 2. Materials and Methods

**Figure 1:**
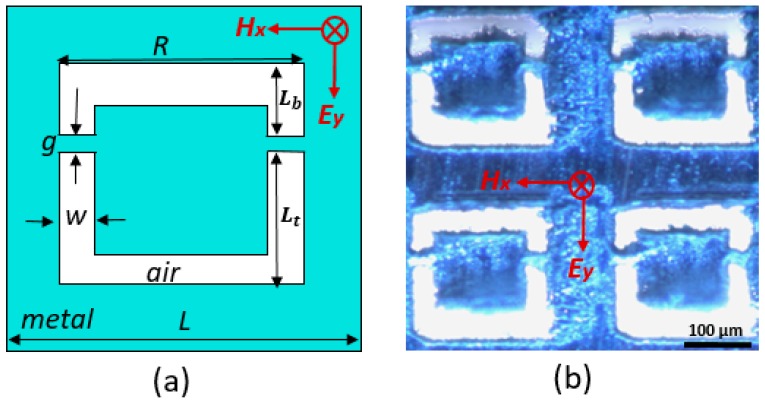
(**a**) Layout of the unit cell of the FCA-SRR with the geometric dimensions *L* = 300 µm, *w* = 25 µm, *R* = 200 µm, *g* = 15 µm. (**b**) Microscopic image of four unit cells of the fabricated structure.

Figure 1a shows the unit cell of the FCA-SRR terahertz metasurface, which was fabricated by laser machining through a 12 µm aluminum layer. Details on the laser cutting process can be found in [47]. Figure 1b shows a microscopic image of the fabricated structure. The obtained cutting linewidth of 8 µm was limited by the focus beam size and the required power to cut the aluminum foil. We use the commercial software Microwave Studio CST to design and simulate the metasurface [50]. The symmetry of the double split-ring resonator is broken by having the two air arms of the resonators such that one is longer than the other. We define the top arm side length *L_b_* and the bottom arm side length *L_t_* as shown in Figure 1a. Moreover, we define the asymmetry parameter as $\delta =[\left(L_{t}-L_{b}\right)/\left(L_{t}+L_{b}\right)]*100\%$. The experimentally investigated structure has an asymmetry parameter of *δ* = 10.9%, which leads to a Fano resonance at 0.435 THz. The metasurface sensor has a size of 9 mm × 9 mm. The sensor was characterized in transmission using terahertz time-domain spectroscopy [51,52]. The terahertz pulses were recorded for a time window of 200 ps, which allows us to obtain a frequency resolution of 5 GHz. The electric (**E**) and magnetic (**H**) fields of the terahertz radiation are both in the plane of the structure, and hence the ***k***-vector is at normal incidence, as shown in Figure 1a.

We produced a free-standing complementary symmetric SRR structure (FCS-SRR) and a free-standing complementary asymmetric SRR structure (FCA-SRR) and excited both with the **E**-field pointing in the *y*-direction. The incident field probed the broken symmetry of the FCA-SRR structure. Hence, the Fano resonance was excited. 

## 3. Sensor Performance Evaluation

**Figure 2:**
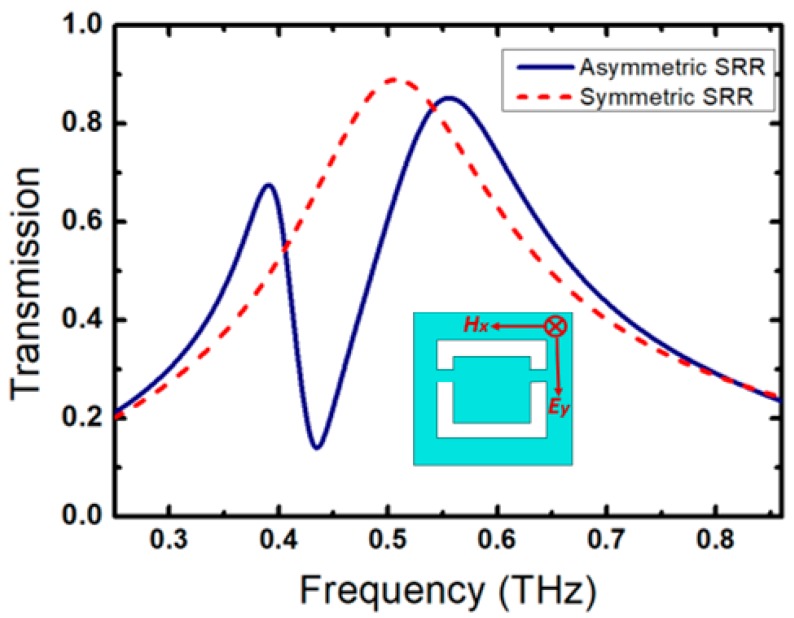
Simulated transmission spectra for the complementary symmetric SRR structure (red dashed-line) and the asymmetric SRR structure (blue solid-line) for an **E**-field orientation in y-direction, as shown in the inset of the figure.

The simulated transmitted amplitude is shown in Figure 2 for a completely symmetric structure (red dashed line) and for an asymmetric structure (blue solid-line). A clear sharp Fano resonance is observed when the symmetry is broken (blue solid line). It shows a *Q*-factor of about 29, which is evaluated by taking the ratio of the resonance frequency *f_0_* = 0.435 THz at a sharp dip and the full width at half maximum bandwidth of (FWHM=Δf=15 GHz), i.e.,$\;Q=f_{0}/\Delta f$.

**Figure 3:**
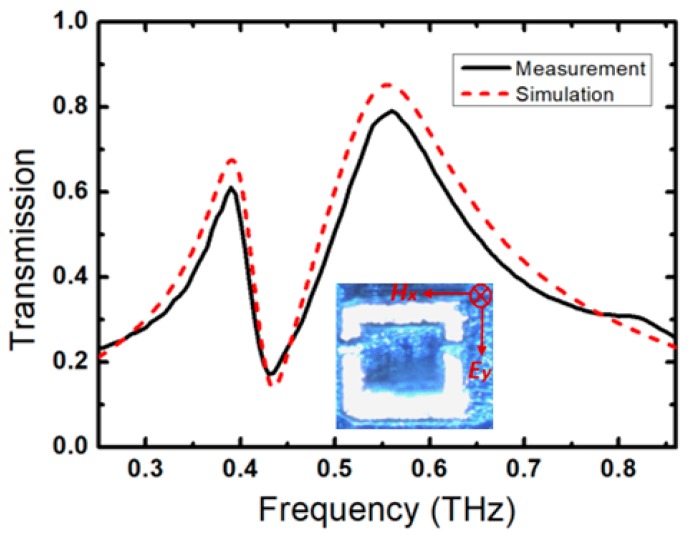
Measured and simulated transmission spectra of FCA-SRR with E-field orientation along y-direction as shown in the inset of the figure when δ = 10.9%.

**Figure 4:**
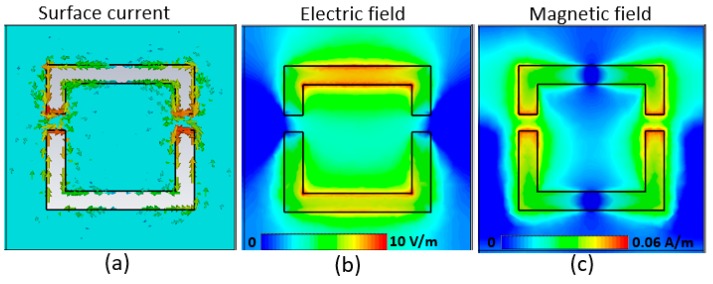
(**a**) Simulated surface current at the resonance frequency of 0.435 THz when the **E**-field is oriented along the y-direction. (**b**) The electric field and (**c**) the magnetic field spatial distributions without analyte for an excitation field amplitude of 1 V/m.

In Figure 3, we present the measured and simulated transmission spectra through the FCA-SRR metasurface. A very good agreement is achieved between the theory and experiment. The little discrepancy in the transmission amplitude is attributed to the roughness of the sensor’s surface, which can be alleviated by reducing the beam size at the focus. Nevertheless, the position of the dip takes place almost at the same frequency. In order to gain insight into the mechanisms causing resonance, we numerically studied the local surface currents, electric field and magnetic field in FCA-SRR. Figure 4 shows a simulated current and magnetic/electric field distribution at the dip resonance frequency of 0.435 THz when the incident electric field is oriented along the *y*-axis. We observe an in-phase current distribution as shown in Figure 4a that leads to a dipole moment suppression and a sharp resonance. The concentration of the current density takes place at the tips of FCA-SRR arms, which corresponds to a strong confinement of the magnetic field as seen in Figure 4c. Moreover, the electric field has a strong distribution in the regions of FCA-SRR, which has a lower surface current density, as seen in Figure 4b. Hence, the confined electric field is highly sensitive to its surrounding material.

**Figure 5:**
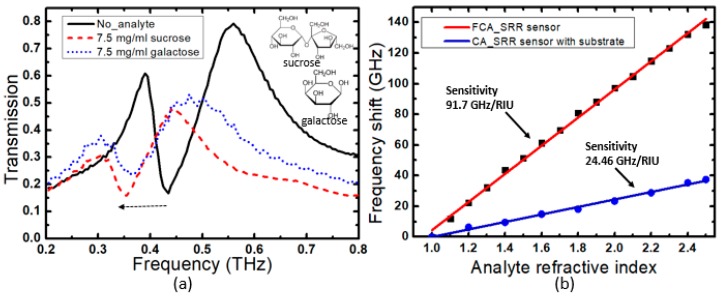
(**a**) Measured amplitude transmission spectra when galactose and sucrose with concentrations of 7.5 mg/mL are deposited on the sensor. (**b**) Frequency shift of the resonant frequency when sweeping the analyte refractive index at an overlayer thickness of 10 µm, for the FCA-SRR sensor (red solid line) and the CA-SRR sensor on top of silicon substrate (blue solid line).

**Figure 6:**
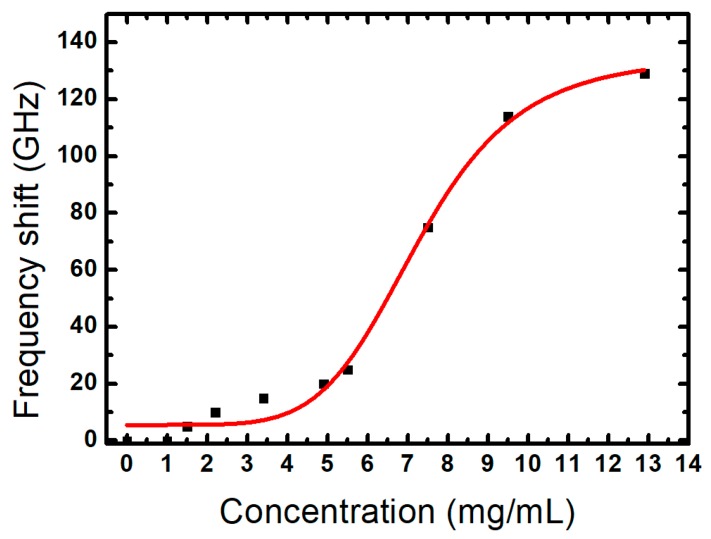
The frequency shift of the resonant frequency of the terahertz transmission as a function of the galactose concentration.

**Figure 7:**
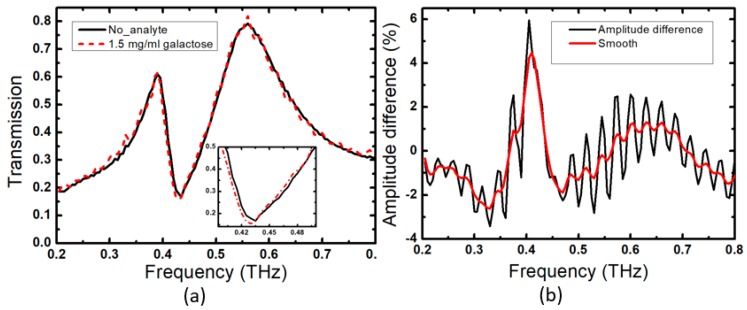
(**a**) Measured amplitude transmission spectra when galactose with concentration of 1.5 mg/mL is deposited on the sensor; the inset shows the response around 0.4 THz; (**b**) The amplitude difference percentage using the ADRT.

The sensitivity of the terahertz sensor was experimentally evaluated in transmission experiments using a terahertz time-domain spectroscopy system. We first studied a bare FCA-SRR structure and then covered it with a layer of galactose and sucrose (with a refractive indexes of *n* = 1.71 and 1.79, respectively). The galactose and sucrose analytes were mixed with water with a concentration of 7.5 mg/mL and then dropped onto the sensor’s surface, where they were left to dry. The deposition of galactose and sucrose analytes induced frequency shifts of 73 and 80 GHz, respectively, which can be seen in Figure 5a where we plot the transmitted amplitude as a function of frequency. Moreover, we evaluated the performance of the FCA-SRR sensor with the same design on top of silicon substrate and studied the effect of the analyte with concentration of 7.5 mg/mL (thickness of about 10 µm) by sweeping the refractive index from 1.1 to 2.5, as shown in Figure 5b. We observe a linear red shift of the Fano resonance with an increase in the refractive index. The linear fit of the frequency shift leads to sensitivities of the free-standing sensor (red solid-line) and the CA-SRR sensor on top of silicon substrate (blue solid-line) of 91.7 GHz/RIU (refractive index unit) and 24.46 GHz/RIU, respectively. The results clearly show that the sensitivity of the free-standing configuration is higher by a factor of 3.75 when compared with its counterpart with the design on the silicon substrate. Furthermore, we experimentally studied how the frequency shifts with varying concentration of galactose between 1 and 13 mg/mL. The results are shown in Figure 6. The black dots correspond to the results of the measurements and the red curve represents an exponential fit. The Fano resonance shifts gradually towards a lower frequency with an increasing concentration of the analyte. Recently, the amplitude difference referencing technique (ADRT) was introduced in [29]. Its use was suggested for very small thickness or very low concentration analytes. Hence, we tested the ADRT based on subtracting the frequency response of the sensor coated by galactose with concentration of 1.5 mg/mL from the frequency response of the sensor without analyte (Figure 7a). The result is shown in Figure 7b, where a very large peak-to-peak difference of 6% has been achieved. Therefore, we can foresee a huge potential of this technique, especially when the frequency shift is quite small for analytes at very low concentration or with a micron or submicron thickness. 

## 4. Conclusions

In conclusion, we investigated a free-standing complementary planar metasurface sensor in the terahertz regime, consisting of a two-dimensional array of FCA-SRR unit cells. The sensor has a frequency sensitivity of 91.7 GHz/RIU. The FCA-SRR sensor was realized by laser beam cutting, which is known for its fast prototyping and low cost. The absence of a substrate eliminates dispersion or losses and offers a high performance of the sensor due to a high interaction between the terahertz confined field and the analyte overlayer. In the future, this free-standing design can offer efficient and cost-effective biosensors.
